# A tool to assess alignment between knowledge and action for health equity

**DOI:** 10.1186/s12889-020-8324-6

**Published:** 2020-02-12

**Authors:** Katrina Marie Plamondon

**Affiliations:** 1 University of British Columbia, 1147 Research Road, Kelowna, BC Canada; 2 Regional Practice Leader, Research & Knowledge Translation, Research Department, Interior Health, Kelowna, BC Canada

**Keywords:** Health equity, Health inequities, Knowledge-to-action, Praxis, Knowledge translation

## Abstract

Advancing health equity is a central goal and ethical imperative in public and global health. Though the commitment to health equity in these fields and among the health professions is clear, alignment between good equity intentions and action remains a challenge. This work regularly encounters the same power structures that are known to cause health inequities. Despite consensus about causes, health inequities persist—illustrating an uncomfortable paradox: good intentions and good evidence do not necessarily lead to meaningful action. This article describes a theoretically informed, reflective tool for assessing alignment between knowledge and action for health equity. It is grounded in an assumption that *progressively more productive action* toward health inequities is justified and desired and an explicit acceptance of the evidence about the socioeconomic, political, and power-related root causes of health inequities. Intentionally simple, the tool presents six possible actions that describe ways in which health equity work *could* respond to causes of health inequities: discredit, distract, disregard, acknowledge, illuminate, or disrupt. The tool can be used to assess or inform any kind of health equity work, in different settings and at different levels of intervention. It is a practical resource against which practice, policy, or research can be held to account, encouraging steps toward equity- and evidence-informed action. It is meant to complement other tools and training resources to build capacity for allyship, de- colonization, and cultural safety in the field of health equity, ultimately contributing to growing awareness of *how* to advance meaningful health equity action.

## Background

Advancing health equity is a central goal and ethical imperative in public and global health. Taking action for health equity is a basic obligation of humanity [1, 2] that has become central to many global governance benchmarks for decades [3–6]. Though the commitment to health equity in these fields and among the health professions is clear [7–9], alignment between good equity intentions and action is a challenge [10–13]. This work regularly encounters the same power structures that are known to *cause* health inequities. Academics, for example, navigate review and funding structures that systematically privilege particular groups [14, 15] and ideologies [16, 17]. In public health practice, efforts to respond to social determinants of health have a tendency to become narrowly focused on behavioural interventions [18–20]. In policy settings, advancing policies to redress imbalances in the distribution of wealth, resources, and power lack traction [21–23]. Regardless of whether health equity work unfolds in practice, policy or research, there seems a common struggle to reconcile an uncomfortable paradox: good intentions and good evidence do not necessarily lead to meaningful action.

In this article, I describe theoretical foundations and provide application examples of a tool developed as part of a series of research studies on promising practices for connecting knowledge to action for health equity [24]. It is grounded in an explicit acceptance of the evidence that demonstrates a causal relationship between health inequities and the distribution of power, resources, and wealth within and between countries [5]. By extension, it also assumes that advancing health equity requires actions that can redistribute power, resources, and wealth. Informed by Paulo Freire’s critical pedagogy [25], principles of cultural safety [26–28], and allyship [29–31], this tool offers reflective questions that can support users to assess how a given action is oriented toward the evidence about causes of health inequities. It could be used to critically reflect on any kind of health equity work, in any setting, at any level of intervention (micro/local, meso/regional-national, macro/global). People working in a variety of settings could use this tool to guide conversations about their intentions, assess alignment between intention and action, and plan for more productive health equity action.

## What is health equity action?

Health equity “means all people (individuals, groups and communities) have a fair chance to reach their full potential and are not disadvantaged by social, economic and environmental conditions” [32]. Achieving health equity requires changing the conditions that create systematic differences in health experiences and outcomes that vary systematically along social gradients [5, 33]. These differences in health experiences and outcomes suffer a wicked tenacity. For example, despite long-standing recognition of the relationship between livelihood and justice [34], vast differences in life expectancy persist between rich and poor populations, both within and between countries [35–38]. The overall global distribution of health risk and disease are “extremely and unacceptably uneven” (Ottersen et al., 2014, p. 630). The life and health trajectories one might enjoy are largely driven by social, environmental, and economic factors [39, 40]—particularly in this epoch of unprecedented health impacts of climate change [41–43]. In essence, the opportunities someone might enjoy for health and well-being are largely determined by structural factors outside of their control, yet caused by human action (or inaction).

Health equity is not a new concept, and actions that restructure the distribution of wealth, resources, and power within and between societies are, at least in part, acts of undoing the harms of centuries-long legacies of oppression and colonization [44–46]. This tool is a means of sparking greater wakefulness to the normative systems, structures, and processes that reinforce unearned advantage and disadvantage in society. It can open receptivity to learning from Indigenous knowledge systems that inherently elevate values of collectivity, caring for society, respect for the responsibilities and limits of humanity’s role in society and in the greater ecosystems in which we live [47]. Despite international consensus on why and how to respond [48], efforts to remediate health inequities are often obstructed by their intersecting causes, including, among others, legacies of colonialism [44, 49, 50], racism [45, 51], structural injustices [52, 53], and failures of neoliberal economic policy [54–57]. Amid these failures, there remain decades-old habits of celebrating health equity work that actually does nothing to remediate the distribution of power, resources, and wealth. Compounding the forces working against alignment between intention, evidence, and action is a persistent preoccupation with bio-behavioural and individualist lenses [19, 58] and socialization to the tolerance of scarcity and suffering of others [59]. These conditions conflate in a collective struggle for integrity and congruence in health equity work. Though the roots are understood, and plausible remediation available, action at all levels remains elusive. Work in this field constantly faces a paradox where our ideals clash with the systems and structures from within which attempts to contribute to a more equitable future are made. For this reason, people involved in health equity work need mechanisms for examining how their efforts align with knowledge about causes of inequities.

### Development & Application of the tool

Recognizing the sociopolitical, economic, and environmental causes of health inequities, this tool was inductively derived to support a series of research studies aimed at identifying promising practices for connecting knowledge with action for health equity [24]. This series began with a scoping review that involved assessing 330 health equity and knowledge translation-relevant publications for signals of integrating evidence about the causes of health inequities [13]. Several signals of integrating evidence were assessed, including citation of key sources of evidence, framing health inequities as having causes that are related to issues of power, and alignment with the World Health Organization’s calls for action on health equity. At the time, many of the tools available for thinking about how to guide health equity action [60–62] were convoluted and provided little practical guidance for reflection about equity options. As the process of reviewing unfolded, it became clear that assessing alignment between knowledge and action required a practical means of assessing a large number of articles, with fidelity, for their *application* of evidence about the causes of health inequities.

The inductive development of the tool involved reflexive practice [63] grounded in: (a) practical experience in global and public health; (b) attention to the generative role of power in perpetuating health inequities; and (c) training in allyship [31, 64] and cultural safety [65]. Though a number of critical theories contributed to my thinking at the time (e.g., [66–68]), Paulo Freire’s critical pedagogy, with its optimism for human agency over the realities we participate in creating, was most influential. Freire proposed that transformative possibilities could be opened through dialogue-based critical examination of issues of power, equity, and resistance-resilience [66, 69]. With Freire’s work in mind, I sought to create something that was focused on identifying *possible* actions while sparking dialogue about the complex issues underlying health inequities. What evolved was a practical tool that could inform critical reflection and dialogue about *how* something—whether it was a project, a research proposal, a policy, or any initiative that aims to advance health equity—was oriented toward the best available evidence about its causes.

The tool was field-tested in capacity-building settings where I was invited to discuss health equity. Quickly understood and applied in different settings (e.g., public health inspectors, population health practitioners, health systems leaders, students, and researchers), it seemed there was promise for this tool to be broadly useful in health equity work at any level of intervention (micro, meso, macro). Feedback at workshops and meetings suggested participants found the tool to be a practical resource against which they could assess current efforts and strategically plan to take steps toward more equity- and evidence-informed action.

### Elements of the tool

**Fig. 1:**
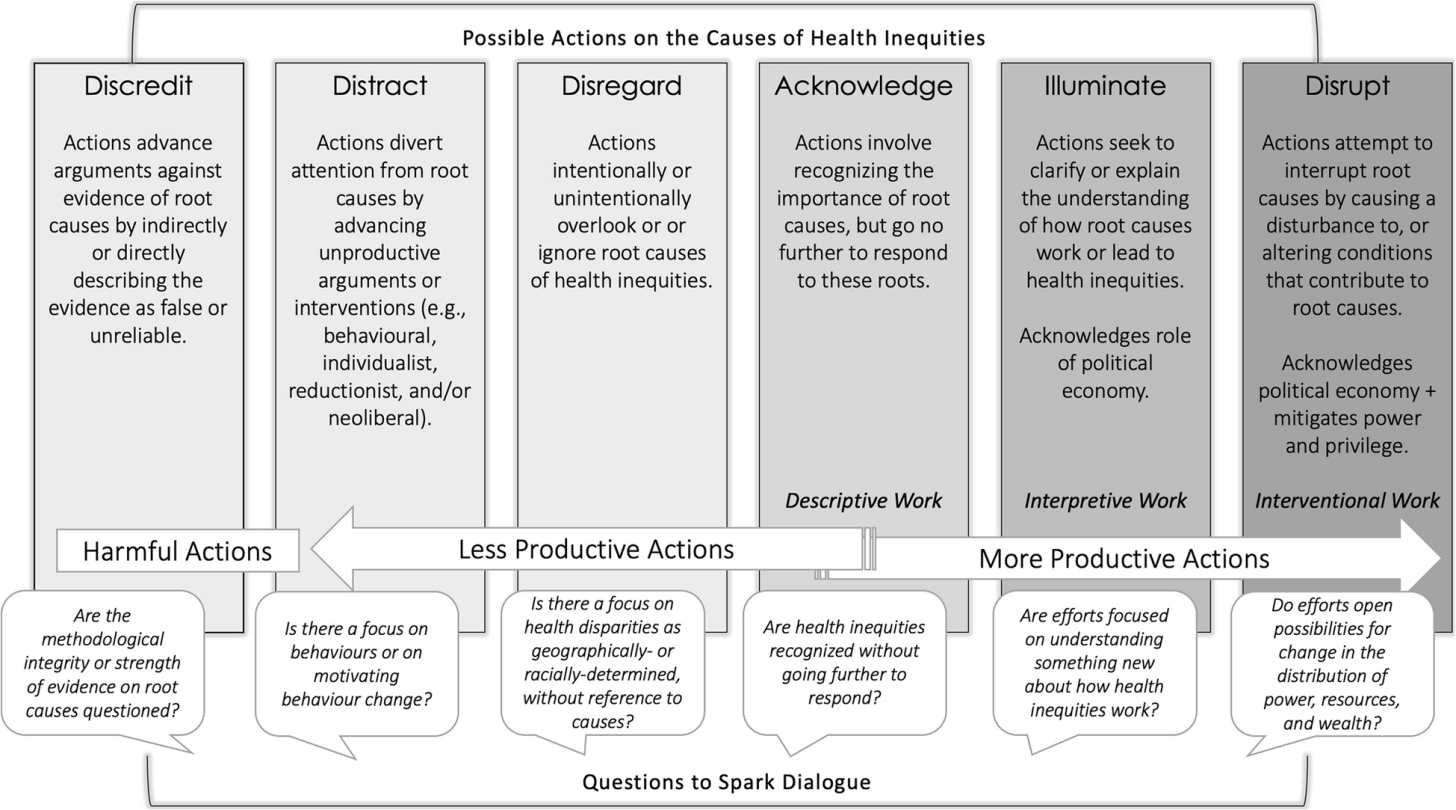
Six possible actions in response to evidence about causes of health inequities. This figure is an adaptation of Table 1 from Katrina M. Plamondon, Joan L. Bottorff, C. Susana Caxaj & Ian D. Graham (2018) The integration of evidence from the Commission on Social Determinants of Health in the field of health equity: a scoping review, Critical Public Health, published online 5th December 2018, DOI: 10.1080/09581596.2018.1551613

The tool, as shown in Fig. 1 [13], adopts an assumption that a range of *more productive* health equity action is justified and desired. It is intentionally simple and direct, offering six actions that describe how an action *could* be directed at the root causes of health inequities. This tool is applicable to any kind of health equity work, either retrospectively or prospectively. For each possible action, the tool offers descriptive language to signal how efforts, through action or inaction, might align with evidence about what causes health inequities. Actions are shown as falling into progressively more or progressively less productive ranges. Among the progressively less productive domains are actions that *disregard* or *distract* from the evidence about what causes health inequities. These actions may be the result of efforts to limit the scope of work or satisfy the demands of power-holders. On the farthest left, actions that *discredit* the legitimacy of causes as something that is known, and may go beyond ‘less productive’, justifying or entrenching systems of oppression or enabling harmful silences.

**Table 1:** Application of the tool to an example at a micro-level

Micro Level Application Actions are carried out at community or local level	Topic Equity Issues in Healthcare Accessibility					
	Examples of Possible Actions					
Point of Application	Discredit	Distract	Disregard	Acknowledge	Illuminate	Disrupt
**Practice** *In an encounter between a public health nurse (PHN) and an Indigenous woman seeking care for an injection-related abscess, the client expresses anger about her treatment at an emergency room, saying she will never return.*	The PHN tells the woman that her recall of the experience was likely inaccurate because of her drug use.	The PHN ignores the woman’s comment and suggests that the she consider treatment for substance use.	The PHN continues to assess the abscess, pretending not to hear the comment.	The PHN continues to assess the abscess, saying, “I know. Many of our clients encounter discrimination in hospitals.”	The PHN provides a safe place for the woman to describe what happened and how it affected her, then invites reflection about the issue of system-wide discrimination in hospitals with colleagues in public health.	Recognizing the damaging impacts of structural violence, the PHN provides a safe place for the woman to describe what happened, how it affected her, and supports the woman to document the encounter in a patient quality care report.
**Policy** *An emergency room manager develops a unit-level policy for triage encounters.*	The policy requires triage staff to approach people appearing to be street-involved with extreme caution because of their violent, unpredictable nature, describing “street-involved” people as “often Aboriginal”.	Arguing street-involved people often leave prior to receiving care, the policy requires staff to offer street-involved persons treatment for substance use during triage.	The policy is silent on discrimination, focusing on procedural rules for what food or clothing staff are permitted to give to street-involved patients.	The policy begins with a purpose statement acknowledging evidence of the impact of racial discrimination on the willingness of street-involved people to seek emergency care, even during critical illness.	The policy expands upon the purpose statement described in ‘acknowledge’, requiring staff to ask and respond to patient safety concerns and access to food and shelter prior to discharge.	The policy focuses on integrating cultural safety in the emergency room through required training, staff.
**Research** *A team of researchers prepare a proposal to identify patterns of healthcare services use among street-involved persons in a community.*	The proposed study identifies genetic patterns among a group of ‘frequent visitors’ to a local emergency room.	The proposed study identifies street-involved people’s healthcare literacy, particularly in understanding when to access alternate services.	The proposed study identifies healthcare service use patterns using postal code data to estimate income by neighborhood, where an absent postal code is categorized as ‘street involved’.	The proposed study identifies healthcare services use among street-involved persons, including asking questions about experiences of racial and poverty discrimination.	The proposed study identifies experiences of structural violence and includes a direct commitment to knowledge translation planning in its design.	The proposed study identifies experiences of the impact of a cultural safety training intervention offered to employees and leadership in hospital settings.

Progressively more productive actions all frame health inequities as having known causes, and range from *acknowledging* to *illuminating* and *disrupting* the systems and structures that unfairly distribute power, resources, and wealth within and between societies. Actions in this range would also be informed by emergent evidence about promising ways to act. For example, action to enact socially-protective policies that are demonstrating success in affecting changes in the social determinants of health, such as the multisector interventions and adoption of universal healthcare coverage in Latin American countries (e.g., [70]). Such action offers the possibility to *disrupt* social and structural drivers of health inequities. In contrast, the consequences of tax policy that incentivizes tobacco production to create jobs may be less productive—and even harmful—because the action distracts from the evidence about how long-term economic impacts are likely to entrench inequities [71]. The former example does something to change the distribution of power, resources, and wealth. The latter, though creating marginal benefits for the few people who gain employment, does something to maintain this distribution—with the greatest benefactor being tobacco companies.

This tool is not intended to minimize the importance of actions that respond directly to urgent needs arising from health inequities. There are reasonable justifications for focusing on work that falls into the distract or dismiss range of actions. They may also have pressing population health needs, bureaucratic barriers, and political challenges that make moving toward more progressive action quite difficult. For example, the opioid crisis requires urgent and downstream interventions to support people in crisis. Yet, if the desire is to stem the tide of opioid overdose, responding only to the end-point consequences will not do anything to resolve the complex social, political, and economic roots of the problems entangled in the crisis. Rather than carrying ‘good’ or ‘bad’ value judgements on different actions, this tool supports evidence- and equity-informed assessment and decision making of public or global health work. If the intention is to advance health equity, then those involved in this work may find utility in being able to identify how much of their work might be positioned to do so.

In Table 1, I provide an example of how the tool might be applied to a micro-level example (equity issues in healthcare accessibility) to show what each of the six actions could look like in a practice, policy, or research setting. These examples demonstrate the multifaceted and more interventional nature of progressively more productive actions. This is because movement toward progressively *more* productive direction advance work that has the potential to redress the complex causes of health inequities. The *acknowledge* domain of action named could be considered either more or less productive.

## Conclusions

Despite international consensus on the evidence about what causes health inequities, much policy, research, and practice related to social determinants of health remains preoccupied with what could be considered ‘symptoms’ rather than causes. Without purposeful attention to collective actions, there is a risk for health equity efforts to slide into a less productive zone that not only maintains inequitable status quos, but also can contribute to normalizing structural inequities. Though it may not be feasible or necessary for all action to *disrupt* root causes of inequities, moving toward progressively more productive health equity action is an ethical imperative. Advancing health equity action requires those involved to weigh their obligations and intentions and make informed decisions about how much of their work will be directed at the best available evidence on the causes of inequities. This tool provides a platform for dialogue about this health equity intention-action alignment. Future steps with this tool include testing and refining the tool and examining the impacts of its application in different settings. At present, this work is beginning to unfold with partners in health systems, municipal, and university settings. As one resource among many that can be used to hold ourselves and others to account, it complements other efforts to build capacity for allyship, de-colonization, and cultural safety in the field of health equity. Different actors have different roles to play in collectively advancing society, including academia, health systems, and governments across sectors. The tool offered here is one way of expanding the methodologies, practices, and languages for more productive action toward a more equitable future.

## Data Availability

Not applicable.
